# Thrombosis and fibrosis: mutually inclusive targets to combat in COVID-19

**DOI:** 10.2144/fsoa-2021-0127

**Published:** 2021-12-16

**Authors:** Mehmet Agirbasli

**Affiliations:** ^1^Department of Cardiology, Medeniyet University Medical School, Istanbul, Turkey

**Keywords:** anticoagulation, COVID-19, fibrosis

SARS-CoV-2 is a novel *Betacoronavirus* that has caused nearly 200 million cases and the COVID-19 pandemic has resulted nearly 5 million deaths world wide [1]. Questions on the long-term efficacy of existing vaccines, and new variants of the virus are harbingers of future waveforms of the disease. Current strategies to manage COVID-19 present unprecedented challenges and an increasing demand for accessible and high quality healthcare globally. The COVID-19 pandemic remains an health emergency particularly in areas of the world with limited access to the healthcare. The challenges include the fact that patients can be completely asymptomatic or experience a mild influenza-like illness, or as in the worst case scenario, patients can present with serious symptoms and lung injury that require hospitalization and intubation. There remains a global urgency to optimize the care and outcomes in critically ill patients.

Lung damage has been suggested as the main cause of morbidity and mortality in COVID-19. The extent of lung involvement in patients with COVID-19 could range from asymptomatic to overt adult respiratory distress syndrome requiring intubation. Our current understanding of the biological basis for the disease course and complications in COVID-19 is rapidly evolving. Further advances in our understanding of pathophysiology of COVID-19 will also enable us to have a closer look into the routinely used medications, such as anticoagulants, in the routine care of these patients. Many pharmaceutical companies already published several randomized controlled trials (RCTs) and started new ones with novel investigational therapeutics. Global recommendation and RCTs are important for effective therapeutic options and strategies, particularly for moderate to severe cases. This editorial reviews the current knowledge about thrombosis, fibrosis and efficacy of anticoagulation regimens in preventing these two mutually inclusive biological processes in COVID-19. The editorial reviews the pathophysiology of COVID-19 to explain the differences in findings between RCTs and potential novel targets for future studies.

## Thrombosis in COVID-19

The autopsy and pathological studies in the lungs of COVID-19 patients report that there are widespread capillary fibrinous microthrombi and fibrin at the microvascular level [2]. Fibrinolysis and proteolytic breakdown of the fibrin in the capillary beds increases the d-dimer levels in the circulation. Elevated d-dimer levels are a biomarker of poor prognosis in COVID-19 [3].

The causes of thrombosis in COVID-19 patients have been investigated extensively over the last couple years. Inflammation has been suggested as the cause of thrombosis in COVID-19. Studies report that intense and widespread inflammation exists in vascular system of COVID-19 patients [4]. There is histologic evidence for SARS-CoV-2-associated endothelitis in specimens obtained from heart, lung, kidney, liver and other tissues in these patients [4].

The other potential cause for thrombosis is the dysfunction of protective proteins in the setting of severe inflammation as a result of COVID-19. Pathophysiology of COVID-19 involves the attachment to the spike protein of the virus to the angiotensin-converting enzyme 2 (ACE2) receptor [5]. A recent meta-analysis with approximately 130,000 public single-cell transcriptome study reports that ACE2-expressing alveolar cells are the primary cellular targets for SARS-CoV-2 infection in the lung [6].

Binding of virus to ACE2 downregulates ACE2. ACE2 is protective and ACE2 also counterbalances the deleterious effects of angiotensin II (Ang II) by converting Ang II to angiotensin 1–7. Loss of ACE2 forms a biochemical pathway that is central in COVID-19 pathophysiology. Loss of ACE2 activates the renin–angiotensin–aldosterone system (RAAS) and exacerbates the harmful effects of Ang II such as thrombosis, tissue proliferation and fibrosis. Activated RAAS is a well-known cardiovascular risk factor in atherosclerosis, kidney and heart failure [7]. RAAS pathway regulates blood pressure, wound healing, extracellular matrix homeostasis, inflammation and hemostasis in metabolic stress [7]. COVID-19, through the elimination of ACE2, leaves the deleterious effects of Ang II unopposed, leading to a constantly activated RAAS. Activated RAAS is thought to be responsible from pulmonary edema, pulmonary fibrin deposition and microvascular thrombosis as displayed in autopsy reports of lungs from COVID-19 patients [2]. Reports show the presence of histologically-proven thrombosis in COVID-19 and clinical observational studies display increased thrombotic events [2,3]. Yet, in most cases pathophysiology and optimal management strategies remain uncertain.

## RCTs evaluating anticoagulants in COVID-19

Administration of proven therapies to patients who are in urgent need and effective utilization of healthcare resources are current and global challenges. Over the last 2 years, our understanding of the pathophysiology of COVID-19 has progressed. We now understand the roles of several biological systems ranging from inflammation, vascular biology, RAAS, hemostasis, coagulation and fibrosis in COVID-19. Yet, there is presently no uniformly and globally accepted treatment algorithms for COVID-19. Protocols widely vary between hospitals and countries (i.e., low vs high income countries). Treatment modalities include supportive measures with oxygen therapy, antivirals, antibiotics for secondary infections, steroids, anticoagulants and immune modulators. Intubated patients who are refractory to treatment often require tocilizumab, and other novel immunomodulatory therapies. There remains an urgent need to develop targeted and proven therapies in COVID-19. A fundamental understanding of the pathophysiology of COVID-19 is required to elucidate the role of current management strategies and therapeutic interventions.

Coagulation disturbances observed in COVID-19, and the potential risk of embolism, have prompted several studies evaluating anticoagulants as a treatment strategy to reduce mortality and morbidity. This is followed by rapidly designed RCTs in patients with confirmed COVID-19 with subsequent plan to develop global recommendations.

Observations report the incidence of pulmonary embolism is increased in critically ill COVID-19 patients [8]. Expert guidelines and local hospital protocols recommend thrombophylaxis with anticoagulants irrespective of risk scores. Routine anticoagulation has been included in the global treatment protocols of COVID-19 [9]. However, this is an indirect assumption in the absence of RCTs results demonstrating that routine anticoagulation strategies reduce the mortality in the hospitalized COVID-19 patients.

The Randomized, Embedded, Multi-factorial Adaptive Platform Trial for Community-Acquired Pneumonia (REMAP-CAP), Therapeutic Anticoagulation; Accelerating COVID-19 Therapeutic Interventions and Vaccines-4 (ACTIV-4) and Antithrombotics Inpatient; and Antithrombotic Therapy to Ameliorate Complications of COVID-19 (ATTACC) are the three large, multicenter RCTs in patients with confirmed COVID-19 infection. These three large platforms examine anticoagulation treatment protocols with subsequent aim to develop global recommendations [10]. The three platforms are similar for the trial design, eligibility criteria, interventions and study end-points which are organ support-free days and in-hospital death [10]. Contrary to the initial assumption, in critically ill patients with COVID-19, therapeutic-dose anticoagulation with unfractionated or low-molecular-weight heparin was not associated with a reduced mortality or morbidity compared with the usual-care with pharmacologic thromboprophylaxis. In fact, trials displayed a probability that therapeutic-dose anticoagulation was inferior to the usual-care thromboprophylaxis [10].

We should look into the reasons for the trial results in view of the complex pathophysiology of COVID-19. Initial observations of increased of pulmonary embolism in COVID-19, as demonstrated by clinical, imaging and laboratory evidence have changed our perceptions of COVID-19. Full-dose anticoagulation regimens in these patients with frequent and multiple comorbidities naturally impose an inherent bleeding risk. Trial results indicate that anticoagulation confers an increased bleeding risk in aggressive protocols for COVID-19 patients.

As a result, the above mentioned RCTs suggest that ‘routine and full-dose anticoagulation’ in the ICU in critically ill COVID-19 patients is not beneficial and in fact can be harmful in some patients [10]. The findings imply the need to test therapies with well conducted RCTs even in the setting of urgent conditions of a global and devastating pandemic.

In this context, we should pay special attention to the observations which indicate that hospitalized patients with COVID-19 display wide physiological and biological heterogeneity [11]. Even though thrombosis is common in COVID-19, certain patients can display increased bleeding risk. As a real challenge to the routine use of anticoagulation strategy, bleeding is observed in 7.6% of COVID-19 patients [12]. To date, unlike thrombosis, bleeding in COVID-19 patients has received less attention in the medical community, but it remains as a real threat particularly for the critically ill patients.

## Dysregulated fibrinolytic system in COVID-19

Given the higher prevalence of the disease in patients with multiple comorbidities, many of the COVID-19 patients are on antiplatelet drugs, and/or anticoagulation for underlying vascular pathologies at baseline. Therefore, RCT results suggest that therapeutic strategies in COVID-19 such as anticoagulation should prompt caution for close monitoring. This is particularly important to avoid complications, since the fibrinolytic system is also dysregulated in critically ill patients with COVID-19 [13]. The plasma concentrations of plasminogen activator inhibitor 1 (PAI-1) and tissue plasminogen activator (t-PA) are crucial in the regulation of the fibrinolytic system, The system of fibrinolysis is also known as plasminogen-plasmin system leading to the formation of active plasmin as the master component. Plasmin is formed by the activation of its inactive precursor plasminogen and remains as the main defense in the body against fibrosis. As discussed earlier, as a fibrin break down product, elevated d-dimer levels are associated with poor prognosis in COVID-19 [3].

Fibrinolytic system is involved in a wide range of biological processes like atherosclerosis, thrombosis, fibrosis, cancer, inflammation, aging, apoptosis and infection [14]. The role of fibrinolytic system components has been extensively investigated in several other pathological conditions prior to COVID-19 [14]. Increased t-PA activity is associated with an increased rate of fibrinolysis and risk of bleeding. On the contrary, elevated plasma PAI-1 levels are associated with thrombosis, atherosclerosis, aging, tumor spread and fibrosis [14].

Prior study reveals that increase in both PAI-1 and t-PA levels are common in critically ill COVID-19 patients [13]. Similar to the coagulation factors, fibrinolytic parameters including PAI-1 and t-PA are significantly dysregulated in COVID-19-related coagulopathy. Increased levels of PAI-1 activity and inhibition of endogenous fibrinolysis can explain the increases in the prevalence of thrombosis and thrombotic complications in COVID-19.

Several other reasons exist for dysregulation of fibrinolysis and its components in COVID-19. Unopposed effects of Ang II in COVID-19 result in increased expression of PAI-1 in endothelial cells [15]. Ang II inhibits fibrinolysis [15]. Ang II and increased PAI-1 result in a hypercoagulable state in critically ill patients with COVID-19. ACE2 acts as a defense mechanism to Ang II effects on endothelial cells. ACE2 converts Ang II to angiotensin 1–7, which decreases the release of PAI-1 from the activated endothelial cells. However, ACE2 is downregulated in COVID-19, as another potential reason for hypercoagulable state in COVID-19 [5,6,15]. Microvascular dysfunction, thrombosis and fibrosis are the potential reasons for accelerated lung injury in COVID-19. The deleterious cascade starts with increased degradation of ACE2 after binding of the virus to ACE2 receptor in the alveolar system [6].

## Thrombosis, fibrosis & COVID-19-related lung injury

Experimental and clinical studies in COVID-19 indicate that interaction between the RAAS and the coagulation system plays a role in the pathophysiology of COVID-19 [6,14,15]. RAAS, kinin–kallikrein (bradykinin) and coagulation systems are activated after SARS-CoV-2 infection, resulting in loss of alveolar cells and fibrosis [6].

The target organs that are commonly affected are the pulmonary and the cardiovascular systems, leading to respiratory failure, heart failure and coagulopathy in critically ill patients. Cytokine storm in during the viral infection can dysregulate defense mechanisms such as ACE2 and endogenous fibrinolysis. Patients experience rapid development of acute inflammatory pulmonary edema as a result of the elevated levels of Ang II, thromboembolism and fibrosis [15]. Anticoagulation or therapeutic strategies; therefore, should take into account the complexity of pathophysiology including several activation and dysregulation of diverse biological processes.

As a result RAAS is activated and fibrinoltic system is inhibited. Both of which set the stage for the thrombotic tendency and fibrosis in COVID-19 [6,14–16]. Diminished fibrinolytic capacity has been described in chronic lung disease prior to COVID-19 pandemia [17,18]. Enhanced PAI-1 activity is associated with poor prognosis in COVID-19 [13]. PAI-1 has a pivotal role in in the lung pathology by enhancement of epithelial–mesenchymal transition and fibrosis [17,18]. Cytokine storm with very high levels of serum pro-inflammatory cytokines significantly stimulate the expression of PAI-1 [19]. Similarly, underlying chronic complex diseases can worsen prognosis in COVID-19 such as metabolic syndrome, obesity, cardiovascular disease, hypertension, renal disease and diabetes. PAI-1 is known to be increased and fibrinolytic system is depressed in all these conditions [15,16].

The role of PAI-1 in COVID-19 has not yet been extensively studied, but building data indicate that this hypothesis merits further attention [19]. Thus, combating thrombosis in COVID-19 extends beyond the use of heparin in hospitalized patients. Elucidating the roles of different biomarkers and modulators in COVID-19 can help the clinician to risk stratify patients who are more susceptible to COVID-19, its complications such as intubation, poor response to vaccines and therapies and have worse outcomes.

## Therapies beyond anticoagulation

Antithrombotic therapy in critically ill patients is often utilized with the common goal of prevention of embolism. Coagulation factors are serine proteases of the coagulation cascade composed of the intrinsic and extrinsic pathways, leading to the formation of thrombin. Increasing evidence suggests that thrombin exerts nonhemostatic cellular effects that are involved in pathophysiological conditions, such as atherosclerosis, inflammation and fibrosis. For example, anticoagulation with enoxaparin, a low molecular-weight heparin, not only prevents development of venous thrombosis in debilitated patients, but also improves survival [20]. Anticoagulant treatment can reduce the progression of fibrosis in end-organ damage such as cirrhosis [20]. However, in the setting of COVID-19 this comes with a risk of bleeding; therefore, outcome studies and RCTs are required to determine the strategies that are associated with improved outcome.

Impaired fibrinolysis and hypercoagulable state contribute to microthrombi and fibrin deposition as the pathogenesis of acute and subacute lung injury in COVID-19. To overcome activated RAAS, and depressed fibrinolysis, fibrinolytic agents and PAI-1 inhibitors are potential new horizons in the management of COVID-19 lung injury.

Several studies utilize tPA, uPA, plasminogen, plasmin and PAI-1 inhibitors. In animal models and experimental studies, activation of fibrinolytic system can reverse lung injury and increase oxygenation with reduction in mortality. Several ongoing trials assess these therapies by investigational new drugs for decreasing lung injury in the setting of COVID-19.

ACE2–SARS-CoV-2 attachment modifies ACE2 catalytic activity, and alters the bradykinin, RAAS and coagulation systems [6]. Studies suggest that bradykinin can mediate the severe lung inflammation in COVID-19, and pharmacological inhibition of bradykinin can benefit patients with severe COVID-19 [21].

For instance, flavonoids including quercetin are water-soluble plant pigments with antioxidant capacity, abundant in fruits and plants [22]. Studies indicate that quercetin has anti-inflammatory and cardiovascular protective effects [22]. The trials are testing isoquercetin in the treatment of COVID-19 [23]. Isoquercetin can be a potential treatment for severe inflammation, as demonstrated in patients with COVID-19.

Similarly, Toll-like receptors (TLRs) are involved in the defense against viral infection and its complications of COVID-19 [24]. RCTs are planned with multiple TLR-modulating agents (TLR agonists/antagonists) in patients with severe COVID-19. Novel strategies and combinations such as anti-IL-6 treatment, in combination with TLR-4 antagonists or TLR-7 agonists in combination with antiviral therapy can present promising targets to be tested in future RCTs [23]. Expanding the pharmacological armamentarium is crucial in the fight against COVID-19 and also to combat future challenges with potential ecological crises in the coming decades.

Under these unprecedented challenges and circumstances, multiple trials for potential drugs and vaccines are ongoing. Similarly, effective vaccines, vaccine equity, planetary health measures to ensure the quality of care globally are ongoing discussions.

The discovery of novel therapeutics against the novel coronavirus can contribute to the global efforts for therapeutics innovation for the COVID-19 care pandemic by anticoagulation, anti-inflammatory and antifibrotic remedies. Omics systems science approaches in the future and harnessing the local and global medicine can help us to develop personalized strategies in COVID-19. As is the case for anticoagulation in COVID-19, the results of ongoing trials will verify the benefit of novel therapeutics and strategies in the care of patients with COVID-19.

## Conclusion

Understanding the balance between coagulation, fibrinolysis and hemostasis will help the clinician to develop optimal approaches to thrombosis prophylaxis in COVID-19. Growing data indicate that thrombosis, fibrinolysis and homeostasis in COVID-19 are complex with subsets of patients that display a tendency for bleeding rather than thrombosis. For instance, recent study indicates that a hyperfibrinolytic state can exist in some COVID-19 patients with poor outcome [13]. Therefore, the coagulopathy of COVID-19 seems to be a complex interplay of several biological systems and factors. The universal recommendations; therefore, need to be selective and cautious to minimize thrombosis versus bleeding risk. Similarly, the anticoagulation recommendations for patients with COVID-19 are in need of anticipatory systems and mechanistic foresight that can detect early signals of safety and efficacy. Furthermore, populational differences exist, and patient outcomes from individual centers can provide the clinician a more informed view. In the absence of robust biomarkers and early mechanistic detection of signals pertaining to thrombosis, it will be difficult to form population-scale recommendations.
